# Psychometric Properties of the Parent-Infant Caregiving Touch Scale

**DOI:** 10.3389/fpsyg.2015.01887

**Published:** 2015-12-15

**Authors:** Artemis Koukounari, Andrew Pickles, Jonathan Hill, Helen Sharp

**Affiliations:** ^1^Department of Biostatistics, Institute of Psychiatry, Psychology and Neuroscience, King's College LondonLondon, UK; ^2^School of Psychology and Clinical Language Sciences, University of ReadingReading, UK; ^3^Department of Psychological Science, Institute of Psychology, Health and Society, University of LiverpoolLiverpool, UK

**Keywords:** mothers, infants, Parent-Infant Caregiving Touch Scale, stroking, early programming

## Abstract

Recent work in animals suggests that the extent of early tactile stimulation by parents of offspring is an important element in early caregiving. We evaluate the psychometric properties of a new parent-report measure designed to assess frequency of tactile stimulation across multiple caregiving domains in infancy. We describe the full item set of the Parent-Infant Caregiving Touch Scale (PICTS) and, using data from a UK longitudinal Child Health and Development Study, the response frequencies and factor structure and whether it was invariant over two time points in early development (5 and 9 weeks). When their infant was 9 weeks old, 838 mothers responded on the PICTS while a stratified subsample of 268 mothers completed PICTS at an earlier 5 week old assessment (229 responded on both occasions). Three PICTS factors were identified reflecting stroking, holding and affective communication. These were moderately to strongly correlated at each of the two time points of interest and were unrelated to, and therefore distinct from, a traditional measure of maternal sensitivity at 7-months. A wholly stable psychometry over 5 and 9-week assessments was not identified which suggests that behavior profiles differ slightly for younger and older infants. Tests of measurement invariance demonstrated that all three factors are characterized by full configural and metric invariance, as well as a moderate degree of evidence of scalar invariance for the stroking factor. We propose the PICTS as a valuable new measure of important aspects of caregiving in infancy.

## Introduction

Recent work in animals suggests that the extent of early tactile stimulation by parents of offspring is an important element in early caregiving. Studies of rodents have shown that tactile stimulation, in the form of maternal licking and grooming of pups, gives rise to epigenetic changes affecting the Glucocorticoid Receptor (GR) gene (Meaney and Szyf, 2005). These changes in turn give rise to changes in HPA axis regulation and levels of fearful behaviors that can persist into adulthood. Extensions of this work has shown that the epigenetic effects are observed even when the tactile stimulation is provided by mechanical brushing rather than natural maternal grooming (Imanaka et al., 2008). Studies of early parental caretaking behavior in humans have, by contrast, typically focused on complex, social, and often multidimensional observational indices, like the construct of maternal sensitivity (Tryphonopoulos et al., 2014), or they required parents to report their beliefs about parenting practices and behaviors (Winstanley and Gattis, 2013) sometimes for quite specific domains of caretaking such as feeding (Hughes et al., 2005, 2012; Thompson et al., 2009) or sleeping, and soothing (Morrell and Cortina-Borja, 2002). Interest in touch within caregiving has largely been limited to promoting early skin to skin contact by parents with premature babies and studies of baby massage interventions. We are not aware of any published work aiming to describe the nature and frequency of naturally occurring parental tactile behaviors toward their young infants in a representative population of new parents.

In adults, research evidence does suggest that tactile stimulation and emotion regulation may be linked. Characteristic patterns of prefrontal cortex and limbic activations have been shown in response to stroking with a pleasant stimulus, such as velvet, contrasted with a neutral or unpleasant stimulus, such as sandpaper (Gordon et al., 2013). Connectivity between these regions is central to effective emotional and behavioral regulation and impaired functioning of each region and failures of connectivity have been hypothesized to underpin conditions such as depression and borderline personality disorder (New et al., 2007). Effects of cortisol on emotion mediated processes such as fear conditioning have also been shown (Merz et al., 2013).

We wished to operationalize the tactile stimulation construct for use within a large mother-infant cohort established and designed to examine the development of infant emotional regulation over time. Parental sensitivity is often assessed as if it was a capacity, being coded directly from brief observation of interaction and often recorded in at least partially standardized settings. However, little is known about how these snapshots of interaction are representative of the typical naturalistic behavior in the infant's everyday environment, something that we considered likely essential for any measure of tactile stimulation. Instead we sought to construct a parent-report measure appropriate for use in infancy with the capacity to provide an overview of both common and rarer behaviors, to do so across different contexts and extended periods of time, and that captured potential confounding effects relating to other forms of mother-infant physical contact and mother-infant separation.

The Parent-Infant Caregiving Touch Scale (PICTS) introduced here is a 12-item parent report scale. Previous work using only a subset of four items from the PICTS reported on carer's early stroking behavior toward their infant and showed that tactile stimulation of infants moderated the effects of prenatal stress on methylation of the GR CpG site (Murgatroyd et al., 2015). Further work demonstrated enduring moderation of prenatal effects, by early stroking, on infant anger proneness and vagal withdrawal at 7 months, and internalizing symptoms at 2.5 years (Sharp et al., 2014). These studies are the first to report the effects of stroking on infant emotion regulation in humans. The fact that parental stroking of infants in early life appears to mimic the observed effects of licking and grooming in rodents on offspring emotion regulation is encouraging. They suggest that this form of early parent-infant caregiving may be a potential target for intervention in the future, if natural levels of stroking behavior are reported to be low. Further work is needed to examine whether associations between stroking and emotion regulation in infants and young children are mediated via HPA axis regulation.

In this paper we describe the full item set of the PICTS, response frequencies and factor structure. We also test whether the factor structure of the scale is stable at each of two time points in early development (5 and 9 weeks) and whether the item composition was invariant during this same period. Examining factor structure answers the question whether the PICTS includes adequate item content representing the obtained factors at 5 and 9 weeks. Examining item invariance allows one to answer the question as to whether reported changes in parental behaviors could be attributed to true change in behavior rather than changes in the psychometric properties of the items during early development. Such features are important as the experience of carers and the caring demands of infants change rapidly in the first few weeks of life. Finally we report on the internal reliability of the PICTS, describe associations with more traditional measures of parenting behavior, and the association with the sex of the infant and parent's age.

## Methods

### Sample

Participants were mothers and infants taking part in the Wirral Child Health and Development Study, a longitudinal UK study of child development. A detailed sample description is given elsewhere (Sharp et al., 2012). In brief, a consecutive series of 1286 first time mothers attending for antenatal care from the sole universal provider of maternity care in the region were recruited. The target post-natal sample available for follow-up with singleton infants was 1233 women. We analyze here data from 838 women who responded on the PICTS at 9 weeks and 268 women in a stratified subsample who, as part of ongoing more intensive assessment, had been previously assessed at 5 weeks (229 responded on both occasions).

All women gave written informed consent at the point of recruitment in the antenatal clinic. Mothers and infants were observed in interaction when their infant was around 7 months of age. Data on maternal sensitivity to distress for 7 and 8 min of mother-infant interaction were available from 164 to 171 women, respectively; and data on maternal sensitivity to non-distress for 7 and 8 min were available from 271 to 270 women, respectively.

Ethical approval for the study was granted by the Cheshire North and West Research Ethics Committee on the 27th June 2006.

### Measures

#### Parent-infant caregiving touch scale

**Figure 1:**
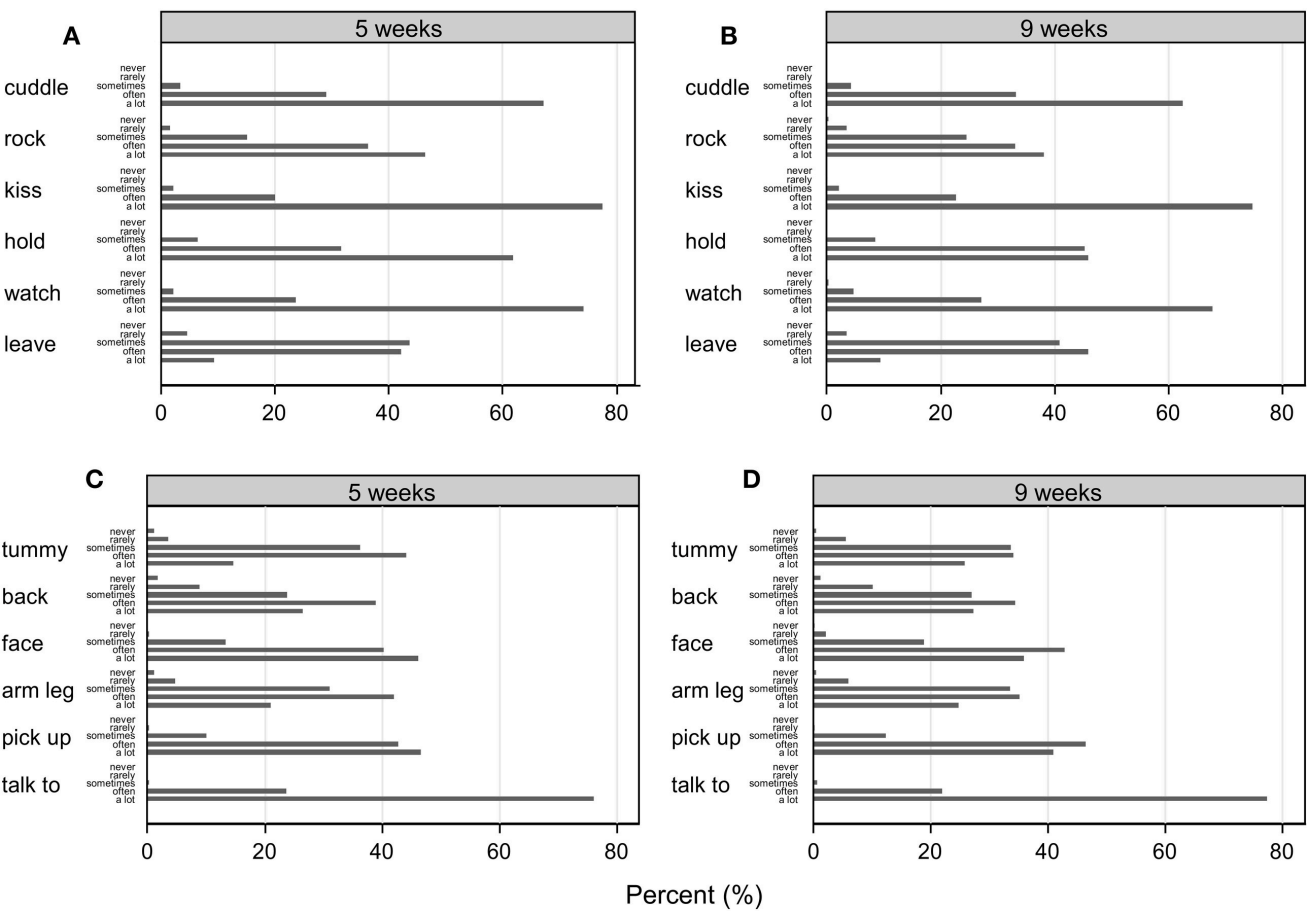
**(A,B)** Represent distribution of items such as cuddling, rocking, kissing, holding, watching, and leave to lie down at 5 and 9 weeks of infants' age, respectively. **(C,D)** Represent distribution of four stroking items on their baby's tummy, back, face, arms and legs and the remaining two items of the questionnaire such as picking up and talking to at 5 and 9 weeks of infants' age, respectively.

Twelve items were constructed to reflect commonly observed parental behaviors in early parent-infant interactions. **Table 2** displays the 12 behaviors examined by the PICTS. Four items assessed tactile stimulation in the form of stroking. These asked how often the mother stroked her baby's back, head, tummy, arms, and legs. Remaining items were selected to reflect various other forms of touch or communication, specifically how often she picked up, cuddled, rocked, kissed, held, talked to, watched or left her baby to lie down. The possible responses for each item consisted of a 5-point Likert scale with levels coded as Never; Rarely; Sometimes; Often; A Lot. Figure 1 presents the observed distributions of the item scores. For analysis some items with rarely scored categories were collapsed to ensure that items had the same number of response categories at both 5 and 9 weeks assessments. The PICTS was completed in paper and pen format on each occasion.

A copy of the PICTS measure is given in Appendix 1 in Supplementary Material.

#### Maternal sensitivity

Maternal sensitivity was assessed at 29 weeks post-natal with a widely used 15 min standard laboratory based procedure (NICHD, 1999). Mothers were asked to play with their infants as they would at home, for 7 min with toys supplied by the mother, and for 8 min with a standard set of toys provided by the experimenter. Maternal sensitivity to distress and non-distress were each rated on a single global 5-point scale, ranging from 1 (not at all characteristic) to 5 (highly characteristic) for each observed interaction period. Sensitivity to distress captured the extent to which the mother responded to her infant's cries, frets or distress in a consistent, timely, and appropriate manner. Sensitivity to non-distress captured the extent to which the mother observed and responded in a well-paced and appropriate manner to her infant's social gestures, expressions, and signals of non-distress. Training on the sensitivity ratings was provided by an investigator from the NICHD Network. Three raters, blind to the other measures, coded sensitivity from video recordings. Each rater achieved good inter-rater reliability for maternal sensitivity on a subset of 30 assessments (ICCs 0.83–0.89).

#### Demographic background

Maternal age in years was recorded at first consent. Social deprivation was measured by the quintiles of the UK Index of Multiple Deprivation (Noble et al., 2004) based on data collected from the UK Census in 2001.

### Statistical analysis

Analyses using the 5-week PICTS data from the intensive subsample were conducted with applied inverse probability weights to account for the stratified sampling of these more intensively assessed families. Thus, estimates and description of both 5 and 9 week data relate to the general population. Descriptive results were obtained from the statistical software SAS version 9.3 (SAS Institute Inc., Cary, NC).

#### Cross-sectional exploratory, confirmatory factor analyses and reliability

We used a Geomin oblique-rotation exploratory factor analysis (Browne, 2001) with Mplus statistical software, Version 7.3 (Muthén and Muthén, 1998–2010) to indicate the broad structure of the 12 ordinal items as completed at 5- and at 9-weeks. We then used a confirmatory factor analysis (CFA) model and selected items by applying the rule of thumb of 0.32 (Tabachnick and Fidell, 2001) as the minimum standardized factor loading which equates to approximately 10% overlapping variance with the other items in that factor. A “cross-loading” item was considered when an item loaded at 0.32 or higher on two or more factors. Factors were considered strong and stable if they had at least three strongly loading items (≥ 0.45). The goodness of fit of the CFA models was assessed by the root mean square error of approximation (RMSEA), accompanied by its associated 90% confidence interval (CI), and by the comparative fit index (CFI).

In the light of criticism of Cronbach alpha for all but the simplest measures (Raykov and Marcoulides, 2011) we report two alternative reliability indices, ordinal alpha (Gadermann et al., 2012) and a non-linear SEM based reliability (Green and Yang, 2009). Ordinal alpha estimates reliability for the sum of the continuous variables that underlie the ordered categorical items and requires tau equivalence among the underlying continuously distributed items as well as uncorrelated errors (Yang and Green, 2015). The non-linear SEM reliability coefficient assesses the reliability for the sum of the observed categorical item scores and allows relaxation of constraints in the factor loadings.

#### Longitudinal factor analysis model and longitudinal invariance

We examined whether the items represented the same underlying constructs over time (i.e., between 5 and 9 weeks), testing for longitudinal invariance following a sequence of steps (or fitting a sequence of longitudinal confirmatory factor models) (Little et al., 2007), by placing a logically ordered series of additional constraints on the initial configural or baseline model to establish weak factorial invariance (i.e., by constraining factor loadings to be equal across measurement occasions) and strong factorial invariance (i.e., by constraining the factor loadings as well as the thresholds of the manifest items on the latent factors to be equal across measurement occasions). Full invariance was deemed to be supported when placing additional constraints on the model did not produce a substantial worsening in model fit. We used WLSMV estimator and the Theta parameterization in which the residual variances for continuous latent response variables of observed categorical outcome variables are allowed to be parameters in the models, but scale factors for continuous latent response variables are not. To compare models, for the WLSMV estimator, the conventional approach of taking the difference between the chi-square values and the difference in the degrees of freedom is not appropriate because the chi-square difference is not distributed as chi-square. We thus used the *DIFFTEST* command in Mplus to evaluate whether a substantial change in model fit occurred as a result of imposing additional equality constraints on particular parameters, as well as the CFI change (ΔCFI), the RMSEA goodness-of-fit statistic, and the degree of overlap in RMSEA confidence intervals between models. A non-significant chi-square difference test and a small CFI (in which a decrease is no greater than 0.01) are considered indicative of invariance (Cheung and Rensvold, 2002). In the event of possible lack of strong factorial invariance we examined the modification indices in order to determine which items failed the strong factorial invariance assumption the most. We then allowed the thresholds of these items to vary freely and reexamined model fit as a test of partial strong factorial invariance.

## Results

### Observed distribution of item scores

Figure 1 shows that while the “never” category is rarely endorsed carers reported variation in the frequency of all the behaviors. Item means appear quite stable from 5 to 9 weeks. Table 1 contains correlations between items which are also quite stable at 5 and 9 weeks.

**Table 1:** **Polychoric correlations between the 12 items at 5 and 9 weeks**.

	**1**	**2**	**3**	**4**	**5**	**6**	**7**	**8**	**9**	**10**	**11**	**12**
I cuddle my baby												
I hold my baby	0.788 0.750											
I kiss my baby	0.695 0.641	0.529 0.409										
I leave my baby to lie down	0.038 0.038	−0.090 −0.126	0.203 0.127									
I pick my baby up	0.731 0.731	0.819 0.913	0.431 0.398	−0.091 −0.126								
I rock my baby	0.713 0.644	0.635 0.603	0.631 0.386	0.055 −0.059	0.632 0.598							
I talk to my baby	0.675 0.734	0.503 0.554	0.581 0.625	0.202 0.148	0.539 0.508	0.481 0.429						
I watch my baby	0.380 0.450	0.486 0.322	0.545 0.458	0.237 0.147	0.313 0.349	0.292 0.320	0.332 0.043					
I stroke my baby's tummy	0.300 0.457	0.265 0.337	0.448 0.412	0.149 0.166	0.251 0.341	0.349 0.519	0.340 0.353	0.292 0.341				
I stroke my baby's back	0.402 0.489	0.247 0.365	0.307 0.366	0.172 0.137	0.234 0.351	0.308 0.484	0.161 0.298	0.269 0.319	0.476 0.645			
I stroke my baby's face	0.278 0.396	0.274 0.282	0.413 0.533	0.114 0.173	0.275 0.295	0.425 0.393	0.327 0.387	0.357 0.396	0.416 0.615	0.242 0.487		
I stroke my baby's arms/legs	0.260 0.429	0.132 0.288	0.327 0.428	0.162 0.143	0.191 0.334	0.312 0.442	0.274 0.307	0.398 0.395	0.583 0.668	0.339 0.581	0.512 0.710	

### Exploratory factor analysis

Although showing a strong major factor, exploratory factor analysis suggested a three-factor solution as the best model superficially similar for both time points. The retained three factors had eigenvalues above 1 (5.311, 1.722, and 1.106 at 5 weeks and 5.714, 1.755, and 1.132 at 9 weeks) and estimated factor loadings are shown in Table 2. The three factors were weakly to moderately correlated (0.253–0.603 at 5 weeks and 0.060–0.597 at 9 weeks).

**Table 2:** **Geomin rotated loadings (5-week EFA, ***n*** = 268; 9-week EFA, ***n*** = 838) for 12 item-scale**.

**Item**	**5-week factor 3 9-week factor 1 *“Stroking”***	**5-week factor 1 9-week factor 3 *“Affective communication”***	**5-week factor 2 9-week factor 2 *“Holding”***
I cuddle my baby	−0.036	**(0.554)**	**(0.544)**
	−0.004	**(0.577)**	**(0.433)**
I hold my baby	0.030	0.291	**0.734**
	−0.004	0.003	**0.973**
I kiss my baby	0.013	**0.841**	0.051
	0.010	**0.822**	−0.075
I leave my baby to lie down	0.034	**(0.457)**	**(**−**0.377)**
	0.113	**(0.328)**	**(**−**0.369)**
I pick my baby up	0.189	−0.003	**0.865**
	0.043	0.000	**0.924**
I rock my baby	0.166	**(0.328)**	**(0.462)**
	0.313	0.053	**0.594**
I talk to my baby	−0.010	**0.595**	0.216
	−0.211	**0.898**	0.038
I watch my baby	0.189	**0.568**	−0.024
	0.067	**0.569**	−0.013
I stroke my baby's tummy	**0.850**	−0.156	−0.001
	**0.756**	0.008	0.313
I stroke my baby's back	**0.520**	−0.011	0.047
	**(0.670)**	−0.042	**(0.357)**
I stroke my baby's face	**0.542**	0.135	0.041
	**(0.562)**	**(0.406)**	−0.035
I stroke my baby's arms/legs	**0.793**	0.015	−0.138
	**0.675**	0.236	0.095

Factor loadings greater than 0.32 are presented in boldface, while cross-loadings greater than 0.32 are presented in parentheses.

### Cross-sectional confirmatory factor analyses

Three different CFA models were initially tested separately for each time point of the study: a unidimensional model; two correlated first-order factors, implying parent-infant caregiving and stroking; and three correlated first-order factors, based on the EFA results. Comparison of the final cross-sectional CFAs allows an assessment of whether the same factorial structure or configural invariance holds during early development.

#### 5-week data

**Figure 2:**
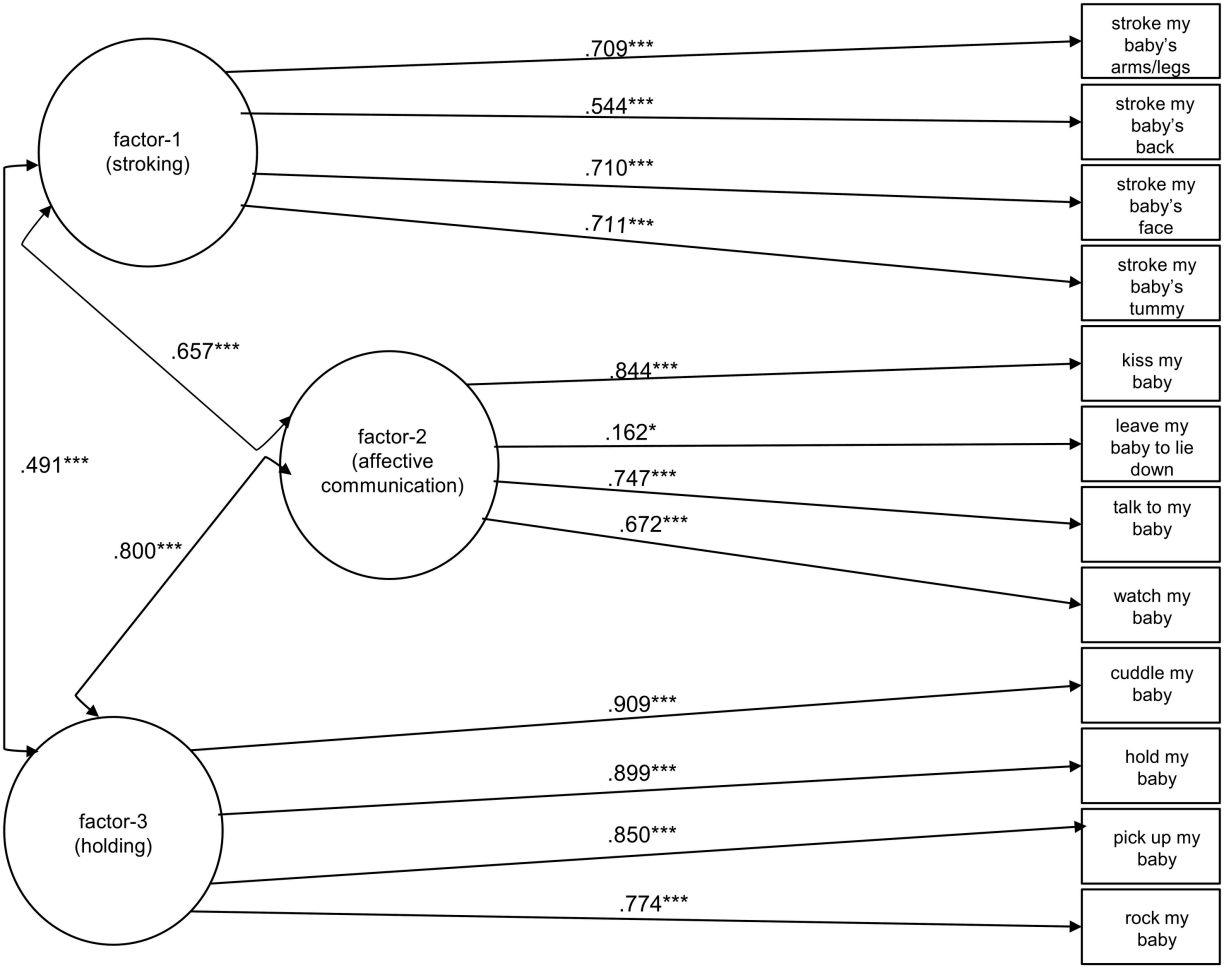
**Standardized estimates of loadings are displayed from the factors (shown in circles) to the 12 ordinal items (shown in squares)**. Arrows between the factors represent the standardized values of their covariances. ^*^P < 0.05; ^***^P < 0.001.

Fit statistics (Table 3) and examination of Modification Indices suggested a 3-factor model as preferred but with a reassignment of the “cuddling” item from Factors 2 to 3. For the selected 3 factor model shown in Figure 2, factor-1 (stroking) is measured by the four maternal stroking items on their baby's face, back, tummy, arms, and legs; factor-2 (affective communication) is measured by the four items: kissing, leave to lie down, talking, and watching; and factor-3 (holding) is measured by the four items: cuddling, holding, picking up, and rocking. The achieved model fit was adequate.

**Table 3:** **Fit statistics for 5-week confirmatory factor analysis of 12 items (***n*** = 268)**.

**Models tested**	**Factor structure**	**χ^2^**	***df***	**CFI**	**RMSEA (95% CI)**
1	1 First-order factor	305.983	54	0.895	0.132 (0.118–0.147)
2^a^	2 First-order correlated factors	152.222	53	0.959	0.084 (0.068–0.099)
3^b^	3 First-order correlated factors	140.851	51	0.963	0.081 (0.065–0.097)
3^c^	3 First-order correlated factors (modified)	122.661	51	0.970	0.072 (0.056–0.089)

a *1st factor is measured by the four maternal stroking items on their baby's face, back, tummy, arms and legs; 2nd factor is measured by the remaining eight items of the questionnaire: cuddling, kissing, leave to lie down, talking, watching, holding, picking up, and rocking*.

b *1st factor is measured by the four maternal stroking items on their baby's face, back, tummy, arms and legs; 2nd factor is measured by the following five items: cuddling, kissing, leave to lie down, talking, and watching; 3rd factor is measured by the following three items: holding, picking up, and rocking*.

c *1st factor is measured by the four maternal stroking items on their baby's face, back, tummy, arms and legs; 2nd factor is measured by the following four items: kissing, leave to lie down, talking, and watching; 3rd factor is measured by the following four items: cuddling, holding, picking up, and rocking*.

#### 9-week data

**Figure 3:**
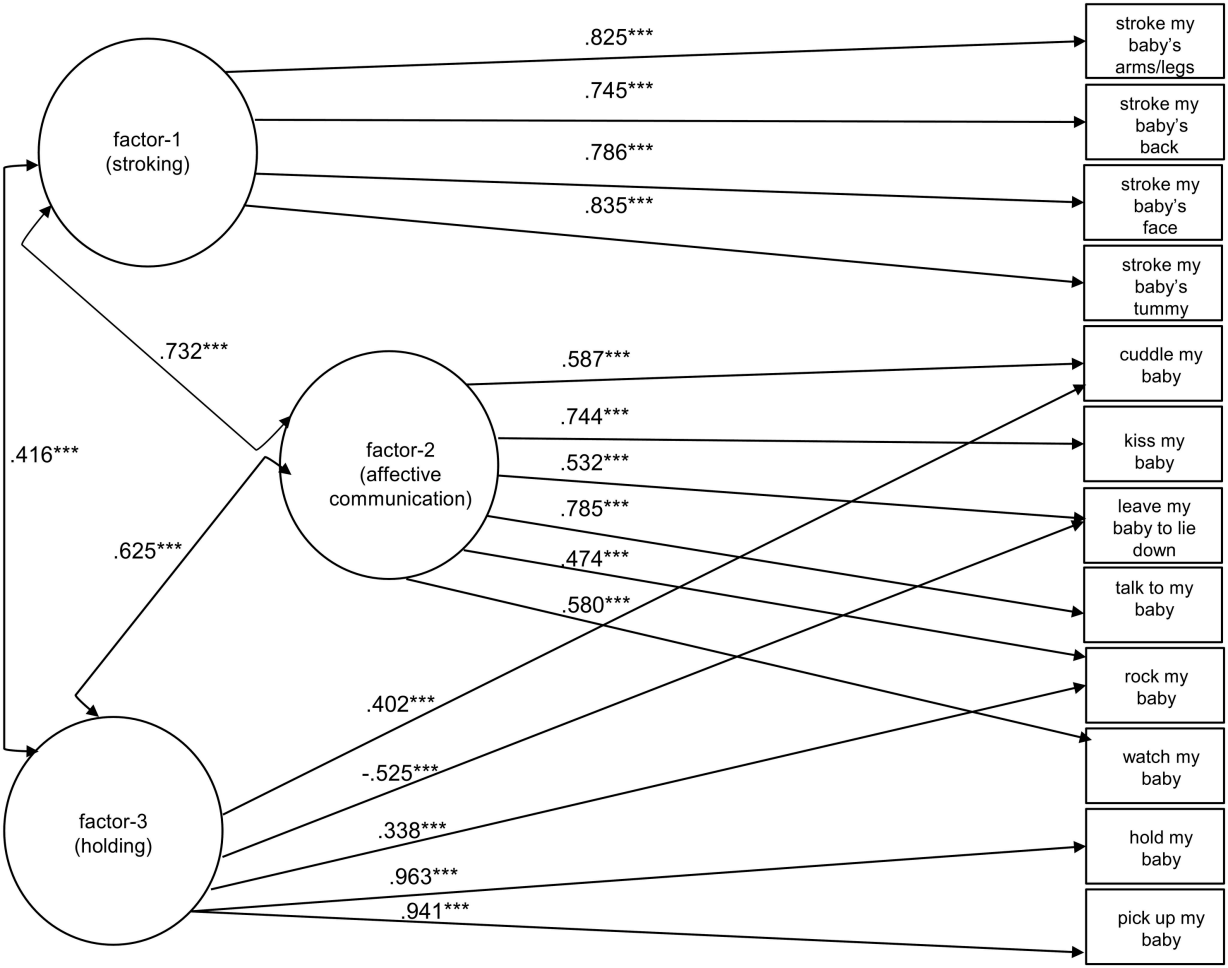
**Standardized estimates of loadings are displayed from the factors (shown in circles) to the 12 ordinal items (shown in squares)**. Arrows between the factors represent the standardized values of their covariances. ^***^P < 0.001.

As shown in Table 4, the configuration selected for the 5-week data did not fit the data from the 9-week data especially well. While the same items loaded on factor 1 and thus configural invariance for stroking applied, the modification indices for the remaining items suggested the inclusion of 2 cross-loaded factors to give the model of Figure 3 in which the “cuddling,” “leave my baby to lie down,” and “rock my baby” items load on both factor-2 (affective communication) and factor-3 (holding). This gave a satisfactory fit (RMSEA less than 0.08).

**Table 4:** **Fit statistics for 9-week confirmatory factor analysis of 12 items (***n*** = 838)**.

**Models tested**	**Factor structure**	**χ^2^**	***df***	**CFI**	**RMSEA fit (95% CI)**
1	1 First-order factor	1562.997	54	0.889	0.183 (0.175–0.190)
2^a^	2 First-order correlated factors	778.927	53	0.947	0.128 (0.120–0.136)
3^b^	3 First-order correlated factors	587.818	51	0.961	0.112 (0.104–0.120)
3^c^	3 First-order correlated factors (modified)	680.979	51	0.954	0.121 (0.113–0.130)
3^d^	3 First-order correlated factors (modified)	448.647	50	0.971	0.098 (0.089–0.106)
3^e^	3 First-order correlated factors (modified)	379.719	49	0.976	0.090 (0.081–0.098)
3^f^	3 First-order correlated factors (modified)	257.322	48	0.985	0.072 (0.064–0.081)

a *1st factor is measured by the four maternal stroking items on their baby's face, back, tummy, arms and legs; 2nd factor is measured by the remaining eight items of the questionnaire: cuddling, kissing, leave to lie down, talking, watching, holding, picking up, and rocking*.

b *1st factor is measured by the four maternal stroking items on their baby's face, back, tummy, arms and legs; 2nd factor is measured by the following five items: cuddling, kissing, leave to lie down, talking, and watching; 3rd factor is measured by the following three items: holding, picking up, and rocking*.

c *1st factor is measured by the four maternal stroking items on their baby's face, back, tummy, arms and legs; 2nd factor is measured by the following four items: kissing, leave to lie down, talking, and watching; 3rd factor is measured by the following four items: cuddling, holding, picking up, and rocking*.

d *Same as model 3^b^ but item rocking is cross-loaded on both factors 2 and 3*.

e *Same as model 3^d^ but also item “cuddling” is cross-loaded on both factors 2 and 3*.

f *Same as model 3^e^ but also item “leave baby to lie down” is cross-loaded on both factors 2 and 3*.

### Reliability

Table 5 contains estimates of reliability based on coefficient alpha as well as alternative reliability indexes including ordinal alpha as well as using the estimates from the CFA models fitted with ordinal items (SAS codes calculating the latter can also be found in Supplementary Material). It is suggested from the Table that Cronbach's alpha is a substantially attenuated estimate of the lower bound of the reliability of the underlying item response variables compared to ordinal alpha and non-linear SEM reliability (Carrol, 1961). Polychoric ordinal alpha and non-linear SEM reliability coefficients indicated very good internal reliability at both 5 and 9 weeks.

**Table 5:** **Estimates of reliability**.

	**5 weeks (*n* = 268)**	**9 weeks (*n* = 838)**
Cronbach's alpha	0.795	0.836
Polychoric ordinal alpha	0.872	0.890
Non-linear SEM reliability coefficient	0.884	0.928

### Associations with infant and maternal characteristics

Variation of the 5-week factor means was examined using a Multiple Indicators Multiple Causes model (Joreskog and Goldberger, 1975). Wald tests suggested no associations of any of the factor means with either the sex of the infant (*p*-values of 0.622, 0.337, and 0.575 for factors 1–3) or with mother's age (*p*-values of 0.824, 0.503, and 0.951 for factors 1–3, respectively).

### Longitudinal confirmatory factor analysis

The cross-sectional results suggest that different factor structures may be necessary at 5 and 9 weeks. We explored this further by examining the impact of forcing a common pattern on the 229 participants with data at both time points applying the 5-week configuration of items to factors of Figure 2 to both 5 and 9 week data (see Supplementary Material Figure S1 for longitudinal confirmatory factor model and for specific Mplus codes). This model exhibiting configural invariance provided a satisfactory fit (χ^2^ = 512.981, *df* = 230, RMSEA = 0.073; 90% Confidence Interval (CI): (0.065–0.082) and CFI = 0.936). The fit was improved when factor loading invariance was imposed (*p* = 0.305 from the chi-square test for difference testing between configural and weak factorial or metric invariance models) which suggests that the same meaning to the latent constructs under study applies at 5 and 9 weeks (χ^2^ = 514.738, *df* = 235, RMSEA = 0.072; 90% CI: 0.065–0.082 and CFI = 0.937). However, a chi-square test for difference testing between metric and scalar invariance models indicated a significantly worse fit with *p* < 0.001 when a scalar invariance model was assumed (i.e., factor loadings and thresholds were constrained to be equal across the 2 time points, residual variances fixed at one at the first time point and free at the second time point, and factor means fixed at zero at the first time point and free at the second time point; χ^2^ = 534.477, *df* = 240, RMSEA = 0.073; 90% CI: (0.065–0.082) and CFI = 0.933) which suggests that the observed differences of mothers' mean responses on the 3 factors between 5 and 9 weeks will be confounded by differences in item-specific thresholds and will not correspond to differences in underlying factor means and thus comparison of the latter during early development is not meaningful. We then explored the issue of partial strong factorial or partial scalar invariance for the stroking factor by constraining only the thresholds of its corresponding items with the exception of the item “stroking baby's face” as modification indices suggested. The fit was then improved (*p* = 0.077 from the chi-square test for difference testing between partial scalar and fully metric invariance models) which suggests that differences in the stroking factor means in early development can either be caused by increases in the thresholds of the item “stroking baby's face” or by true reduction in the stroking factor during this period (χ^2^ = 518.035, *df* = 237, RMSEA = 0.072; 90% CI: (0.064–0.080) and CFI = 0.936).

### Discriminant validity

Do these parental caregiving factors represent something other than more widely adopted observer measures of maternal sensitivity? The 247 ratings made from maternal sensitivity at 7 months of age when the infant was not distressed (NICHD, 1999) correlated only −0.027 (*p* = 0.671) with factor 1 (stroking), −0.018 (*p* = 0.779) with factor 2 (affective communication) and −0.020 (*p* = 0.756) with factor 3 (holding). Sensitivity exhibited when the infant was distressed, available only for the sub-sample of 120 infants of who became distressed, were similarly unrelated (*r* = 0.092, *p* = 0.320 with factor 1; *r* = 0.129, *p* = 0.158 with factor 2; *r* = 0.119, *p* = 0.194 with factor 3).

## Discussion

The PICTS is a parent-report questionnaire that assesses the frequency of 12 behaviors common in the care of human infants but whose selection had been informed by work in animal behavior. Data from this scale for infants at 5-weeks and 9-weeks of age indicated two factors being clearly evident in factor structures at both 5 and 9 weeks. A third factor was less clearly defined. The first clear factor (factor 1) related to carers stroking their infant, and has already been shown to give rise to changes in the epigenome, physiological response and behavior of rodents and human infants (Sharp et al., 2012). The second clear factor (factor 3) was made up of holding and other behaviors that involve this close physical contact with the infant. Holding with skin-to-skin contact in early life has received considerable attention, particularly for infants thought to be at risk of difficulties in regulation, such as kangaroo care for pre-term infants. Commonly promoted as a therapy, the evidence for its beneficial effect is mixed. For example, randomized controlled trials on preterm infants (Neu et al., 2014) find no effects on cortisol expression while other studies (Mitchell et al., 2013) do find effects on bradychardia and oxygen desaturation. Although moderate to strong correlations were estimated across factors at both time points of interest (see Figures 2, 3) maternal tactile stimulation in the form of stroking behavior clearly formed a defined factor, distinct from other forms of “holding” touch assessed using the PICTS measure. Future work will need to determine the relative role of parental stroking vs. holding behaviors in promoting healthy infant development; extending findings which so far suggest stroking moderates prenatal effects of stress on anger proneness and vagal withdrawal at 7 months, internalizing symptoms at 2.5 years (Sharp et al., 2014) and internalizing and disruptive behavior problems at 3.5 years.

We did not find a wholly stable psychometry for the PICTS over 5 and 9-week assessments. While the stroking factor shows partial strong factorial or partial scalar invariance, the item assignment for non-stroking factors (affective communication and holding) change a little from 5 to 9 weeks. It may be that the 3 cross loading items (rocking, cuddling and the negatively loading leave to lie down), represent aspects of caregiving that serve both functions as the infant develops. We note that the item “Leave my baby to lie down” loaded negatively onto the holding factor, which is consistent with the item indexing a lack of physical contact between the dyad, whereas it loaded positively onto the affective communication factor. Although at first this appears to be a contradictory finding, this does makes sense developmentally, since leaving the baby to lie down on occasion, like rocking and cuddling, can be construed as positive strategy which promotes affect regulation in the young infant. In addition, correlations between items at both 5 and 9 weeks showed that those mothers who report more holding, report less of leaving their baby to lie down and vice versa. Future work might usefully further explore the stability in psychometry of the PICTS scale over longer periods in infancy.

Maternal reports for boy and girl infants showed similar means for all factors at 5-weeks and unlike many measures of parenting, the factors also showed no association with maternal age. The fact that scores on the PICTS factors were unrelated to more complex, “gold standard” early measures of caregiving quality such as maternal sensitivity rated from observed mother-infant interaction at 7-months, confirms the distinctiveness of the PICTS constructs. As such we believe the PICTS to be a valuable new measure which will enable us to more fully characterize the infant's early parenting environment and to further investigate what specific functions these types of caretaking behaviors serve in early infant development. It should be noted that further work is required to confirm the properties of the PICTS in non-Caucasian and non-UK populations as well as investigation of further convergent validity tests of the PICTS dimensions and their associations with measures of personality and their nomological network such as juvenile delinquency, childhood psychopathology, school performance and intelligence, and finally socioeconomic status The PICTS is freely available on request.

## Author contributions

AK, AP, JH, and HS wrote the paper. AK and AP conducted the data analysis. AP, JH, and HS designed and conducted the study.

## Funding

The Wirral Child Health and Development Study (WCHADS) was funded by the UK Medical Research Council (MRC; G0400577). AK and AP were supported in part by a grant funded by the National Institute for Health Research (NIHR) Biomedical Research Centre at South London and Maudsley National Health System (NHS) Foundation Trust and King's College London. The views expressed are those of the author(s) and not necessarily those of the NIHR, the NHS, or the UK Government Department of Health.

### Conflict of interest statement

The authors declare that the research was conducted in the absence of any commercial or financial relationships that could be construed as a potential conflict of interest.
