# Supplementary material for: Psychometric Properties of the Parent-Infant Caregiving Touch Scale
Source: Front Psychol. 2015 Dec 15;6:1887. doi: 10.3389/fpsyg.2015.01887 (PMC4678235; doi:10.3389/fpsyg.2015.01887)
Supplement: Supplementary file 1 [file DataSheet1.DOCX]

**Statistical Analysis**

**Cross-sectional Exploratory, Confirmatory Factor Analyses**

For the estimation of the CFAs we used options COMPLEX TYPE of analysis and WEIGHTS where necessary, using the weighted least square estimator (e.g. WLSMV estimator) which uses PROBIT functions to link the observed ordinal indicators to their underlying latent variables/factors).

Model modification indices (MI), being measures of predicted decrease in chi-square values if a single parameter is freed and the model is re-estimated, guided the decisions to change the models and improve fit. Since modification indices are sensitive to sample size, the expected parameter change (EPC) value for each modification index, as a direct estimate of the size of misspecification for fixed parameters, was also examined.

**Longitudinal Factor Analysis Model and Longitudinal Invariance**

Insert Figure S1 here

**Legend for Figure S1:** *Longitudinal Confirmatory Factor Model Path Diagram testing different forms of invariance*

**Computer codes**

**Example Mplus syntax for configural invariance**

DATA: FILE IS PHASE45NN.DAT;

VARIABLE: NAMES ARE id wt12 p4c p4h p4k p4l p4pu p4r p4stral p4strb p4strf p4strt p4t p4w

p5stral p5strb p5strf p5strt p5c p5h p5k p5l p5pu p5r p5t p5w;

MISSING=ALL(-9);

CATEGORICAL ARE p4c p4h p4k p4l p4pu p4r p4stral p4strb p4strf p4strt p4t p4w

p5stral p5strb p5strf p5strt p5c p5h p5k p5l p5pu p5r p5t p5w;

USEVARIABLES p4c p4h p4k p4l p4pu p4r p4stral p4strb p4strf p4strt p4t p4w

p5stral p5strb p5strf p5strt p5c p5h p5k p5l p5pu p5r p5t p5w;

weight is wt12;

CLUSTER IS ID;

ANALYSIS:

TYPE = COMPLEX;

PARAMETERIZATION = THETA;

ESTIMATOR = WLSMV;

MODEL:

!Model 1: Configural Longitudinal Invariance

!Factor Loadings

F41 BY p4stral p4strb p4strf p4strt@1;

F42 BY p4l p4t p4w p4k@1;

F43 BY p4c* p4h p4pu@1 p4r;

F51 BY p5stral* p5strb p5strf p5strt@1;

F52 BY p5l p5t p5w p5k@1;

F53 BY p5c* p5h p5pu@1 p5r;

!Thresholds - free across time but for identification reasons constrain one threshold per latent response variate to be invariant. In addition, for each of the *r* reference variates, require a second threshold to be invariant.

[p4stral$1] (21); [p4strb$1] (22); [p4strf$1] (24)

[p4strt$1](25); [p4strt$2](26); ! anchor item identifying factor mean

[p5stral$1] (21); [p5strb$1] (22); [p5strf$1] (24);

[p5strt$1] (25); [p5strt$2](26); ! anchor item identifying factor mean

[p4l$1] (38); [p4t$1] (35); [p4w$1] (34);

[p4k$1] (47); [p4k$2] (39); ! anchor item identifying factor mean

[p5l$1] (38); [p5t$1] (35); [p5w$1] (34);

[p5k$1] (47); [p5k$2] (39); ! anchor item identifying factor mean

[p4c$1] (41); [p4h$1] (42); [p4pu$1] (43);

[p4pu$2] (44); [p4r$1] (45);

[p5c$1] (41); [p5h$1] (42); [p5pu$1] (43);

[p5pu$2] (44); [p5r$1] (45);

!Residual variances fixed @ 1 @1st time point and free @ the 2nd

p4c@1 p5c*1; p4h@1 p5h*1;

p4l@1 p5l*1; p4pu@1 p5pu*1;

p4r@1 p5r*1; p4stral@1 p5stral*1;

p4strb@1 p5strb*1; p4strf@1 p5strf*1;

p4strt@1 p5strt*1; p4t@1 p5t*1;

p4w@1 p5w*1; p4k@1 p5k*1;

!Factor variances and covariances (all free now)

F41* F42* F43* F51* F52* F53*;

F41 WITH F42*; F41 WITH F43*;

F42 WITH F43*; F51 WITH F52*;

F51 WITH F53*; F52 WITH F53*;

F41 WITH F51*; F41 WITH F52*;

F41 WITH F53*; F42 WITH F51*;

F42 WITH F52*; F42 WITH F53*;

F43 WITH F51*; F43 WITH F52*;

F43 WITH F53*;

!Factor means fixed at 0 both time points

[F41@0 F42@0 F43@0 F51@0 F52@0 F53@0];

!Residual covariances for same indicator across time

p4c WITH p5c; p4h WITH p5h;

p4l WITH p5l; p4pu WITH p5pu;

p4r WITH p5r; p4stral WITH p5stral;

p4strb WITH p5strb; p4strf WITH p5strf;

p4strt WITH p5strt; p4t WITH p5t;

p4w WITH p5w; p4k with p5k;

OUTPUT: SAMPSTAT TECH4 STDYX;

SAVEDATA: DIFFTEST IS DERIV1.DAT;

**Example Mplus syntax for metric invariance**

DATA: FILE IS PHASE45NN.DAT;

VARIABLE: NAMES ARE id wt12 p4c p4h p4k p4l p4pu p4r p4stral p4strb p4strf p4strt p4t p4w p5stral p5strb p5strf p5strt p5c p5h p5k p5l p5pu p5r p5t p5w;

MISSING=ALL(-9);

CATEGORICAL ARE p4c p4h p4k p4l p4pu p4r p4stral p4strb p4strf p4strt p4t p4w p5stral p5strb p5strf p5strt p5c p5h p5k p5l p5pu p5r p5t p5w;

USEVARIABLES p4c p4h p4k p4l p4pu p4r p4stral p4strb p4strf p4strt p4t p4w p5stral p5strb p5strf p5strt p5c p5h p5k p5l p5pu p5r p5t p5w;

weight is wt12;

CLUSTER IS ID;

ANALYSIS: TYPE = COMPLEX;

PARAMETERIZATION = THETA;

ESTIMATOR = WLSMV;

DIFFTEST IS DERIV1.DAT;

MODEL:

!Model 2: Metric Longitudinal Invariance

!Now constrain Factor Loadings at 2 time points

F41 BY p4stral*(1) p4strb (2) p4strf (3) p4strt@1 (4);

F42 BY p4l (6) p4t (7) p4w (8) p4k@1 (46);

F43 BY p4c* (9) p4h (10) p4pu@1(11) p4r (12);

F51 BY p5stral* (1) p5strb (2) p5strf (3) p5strt@1 (4);

F52 BY p5l (6) p5t (7) p5w (8) p5k@1 (46);

F53 BY p5c* (9) p5h (10) p5pu@1 (11) p5r (12);

!Thresholds - free across time but for identification reasons constrain one threshold per latent response variate to be invariant. In addition, for each of the *r* reference variates, require a second threshold to be invariant.

[p4stral$1] (21); [p4strb$1] (22); [p4strf$1] (24);

[p4strt$1](25); [p4strt$2](26); ! anchor item identifying factor mean

[p5stral$1] (21); [p5strb$1] (22); [p5strf$1] (24);

[p5strt$1] (25); [p5strt$2](26); ! anchor item identifying factor mean

[p4l$1] (38); [p4t$1] (35); [p4w$1] (34);

[p4k$1] (47); [p4k$2] (39); ! anchor item identifying factor mean

[p5l$1] (38); [p5t$1] (35); [p5w$1] (34);

[p5k$1] (47); [p5k$2] (39); ! anchor item identifying factor mean

[p4c$1] (41); [p4h$1] (42); [p4pu$1] (43); [p4pu$2] (44); [p4r$1] (45);

[p5c$1] (41); [p5h$1] (42); [p5pu$1] (43); [p5pu$2] (44); [p5r$1] (45);

!Residual variances fixed @ 1 @ the 1st time point and free @ the 2nd

p4c@1 p5c*; p4h@1 p5h*; p4l@1 p5l*; p4pu@1 p5pu*;

p4r@1 p5r*; p4stral@1 p5stral*; p4strb@1 p5strb*;

p4strf@1 p5strf*; p4strt@1 p5strt*; p4t@1 p5t*;

p4w@1 p5w*; p4k@1 p5k*;

!Factor variances and covariances (all free now)

F41* F42* F43* F51* F52* F53*;

F41 WITH F42*; F41 WITH F43*;

F42 WITH F43*; F51 WITH F52*;

F51 WITH F53*; F52 WITH F53*;

F41 WITH F51*; F41 WITH F52*;

F41 WITH F53*; F42 WITH F51*;

F42 WITH F52*; F42 WITH F53*;

F43 WITH F51*; F43 WITH F52*;

F43 WITH F53*;

!Factor means fixed at 0 at one time point and free at the 2nd

[F41@0 F42@0 F43@0 F51* F52* F53*];

!Residual covariances for same indicator across time

p4c WITH p5c; p4h WITH p5h;

p4l WITH p5l; p4pu WITH p5pu;

p4r WITH p5r; p4stral WITH p5stral;

p4strb WITH p5strb; p4strf WITH p5strf;

p4strt WITH p5strt; p4t WITH p5t;

p4w WITH p5w; p4k WITH p5k;

OUTPUT: SAMPSTAT TECH4 STDYX;

**Example Mplus syntax for scalar invariance**

DATA: FILE IS PHASE45NN.DAT;

VARIABLE: NAMES ARE id wt12 p4c p4h p4k p4l p4pu p4r p4stral p4strb p4strf p4strt p4t p4w p5stral p5strb p5strf p5strt p5c p5h p5k p5l p5pu p5r p5t p5w;

MISSING=ALL(-9);

CATEGORICAL ARE p4c p4h p4k p4l p4pu p4r p4stral p4strb p4strf p4strt p4t p4w p5stral p5strb p5strf p5strt p5c p5h p5k p5l p5pu p5r p5t p5w;

USEVARIABLES p4c p4h p4k p4l p4pu p4r p4stral p4strb p4strf p4strt p4t p4w p5stral p5strb p5strf p5strt p5c p5h p5k p5l p5pu p5r p5t p5w;

weight is wt12;

CLUSTER IS ID;

ANALYSIS:

DIFFTEST IS DERIV.DAT;

TYPE = COMPLEX;

PARAMETERIZATION = THETA;

ESTIMATOR = WLSMV;

ITERATIONS=12000;

MODEL:

!Model 3: Scalar Longitudinal Invariance

!Constrain Factor Loadings

F41 BY p4stral*(1) p4strb (2) p4strf (3) p4strt@1 (4);

F42 BY p4l (6) p4t (7) p4w (8) p4k@1 (49);

F43 BY p4c* (9) p4h (10) p4pu@1(11) p4r (12);

F51 BY p5stral* (1) p5strb (2) p5strf (3) p5strt@1 (4);

F52 BY p5l (6) p5t (7) p5w (8) p5k@1 (49);

F53 BY p5c* (9) p5h (10) p5pu@1 (11) p5r (12);

!Constrain Thresholds

[p4stral$1] (21); [p4stral$2] (22);

[p4strb$1] (24); [p4strb$2] (25);

[p4strf$1] (27); [p4strf$2] (28);

[p4strt$1](30); [p4strt$2](31);

[p5stral$1] (21); [p5stral$2] (22);

[p5strb$1] (24); [p5strb$2] (25);

[p5strf$1] (27); [p5strf$2] (28);

[p5strt$1] (30); [p5strt$2](31);

[p4l$1] (32); [p4l$2] (33);

[p4w$1] (35); [p4t$1] (36);

[p5l$1] (32); [p5l$2] (33);

[p5w$1] (35); [p5t$1] (36);

[p4c$1] (41); [p4c$2] (46);

[p4h$1] (42); [p4h$2] (47);

[p4pu$1] (43); [p4pu$2] (44);

[p4r$1] (45); [p4r$2] (48);

[p5c$1] (41); [p5c$2] (46);

[p5h$1] (42); [p5h$2] (47);

[p5pu$1] (43); [p5pu$2] (44);

[p5r$1] (45); [p5r$2] (48);

!Residual variances fixed @ 1 @ the 1st time point and free @ the 2nd

p4c@1 p5c*; p4h@1 p5h*;

p4l@1 p5l*; p4pu@1 p5pu*;

p4r@1 p5r*; p4stral@1 p5stral*;

p4strb@1 p5strb*; p4strf@1 p5strf*;

p4strt@1 p5strt*; p4t@1 p5t*;

p4w@1 p5w*; p4k@1 p5k*;

!Factor variances and covariances (all free now)

F41* F42* F43* F51* F52* F53*;

F41 WITH F42*; F41 WITH F43*;

F42 WITH F43*; F51 WITH F52*;

F51 WITH F53*; F52 WITH F53*;

F41 WITH F51*; F41 WITH F52*;

F41 WITH F53*; F42 WITH F51*;

F42 WITH F52*; F42 WITH F53*;

F43 WITH F51*; F43 WITH F52*;

F43 WITH F53*;

!Factor means fixed at 0 at one time point and free at the 2nd

[F41@0 F42@0 F43@0 F51* F52* F53*];

!Residual covariances for same indicator across time

p4c WITH p5c; p4h WITH p5h;

p4l WITH p5l; p4pu WITH p5pu;

p4r WITH p5r; p4stral WITH p5stral;

p4strb WITH p5strb; p4strf WITH p5strf;

p4strt WITH p5strt; p4t WITH p5t;

p4w WITH p5w; p4k WITH p5k;

OUTPUT: SAMPSTAT TECH4 STDYX;

**SAS code modified as by Yang, Y., & Green, S. B. (2015)**

/***********the following part is for the 5 weeks data*******************/

**proc** **iml**;

RESET fuzz;

THRESH={

-**2.874** -**1.850** -**0.466** **0**,

-**1.554** -**0.335** **0** **0**,

-**2.955** -**2.072** -**0.829** **0**,

-**1.626** -**0.057** **1.430** **0**,

-**2.641** -**1.246** **0.027** **0**,

-**2.163** -**0.985** **0.041** **0**,

-**2.264** -**1.569** -**0.320** **0.727**,

-**2.109** -**1.309** -**0.412** **0.612**,

-**2.641** -**1.085** **0.091** **0**,

-**2.186** -**1.733** -**0.203** **1.111**,

-**0.768** **0** **0** **0**,

-**2.084** -**0.697** **0** **0**

};

LOAD={

**0** **0** **0.909**,

**0** **0** **0.899**,

**0** **0.844** **0**,

**0** **0.162** **0**,

**0** **0** **0.850**,

**0** **0** **0.774**,

**0.709** **0** **0**,

**0.544** **0** **0**,

**0.710** **0** **0**,

**0.711** **0** **0**,

**0** **0.747** **0**,

**0** **0.672** **0**

};

/***********this is to remember the order of the items

/* 1 P4C

2 P4H

3 P4K

4 P4L

5 P4PU

6 P4R

7 P4STRAL

8 P4STRB

9 P4STRF

10 P4STRT

11 P4T

12 P4W

/*************************************************************/

FACCOR={**1** **0.657** **0.491**,**0.657** **1** **0.800**,**0.491** **0.800** **1**};

/******POLY matrix from Mplus output******/

POLY={

**1** **0.803** **0.683** **0.059** **0.740** **0.697** **0.250** **0.365** **0.326** **0.225** **0.665** **0.446**,

**0.803** **1** **0.574** -**0.037** **0.830** **0.661** **0.150** **0.236** **0.326** **0.259** **0.507** **0.509**,

**0.683** **0.574** **1** **0.246** **0.458** **0.609** **0.338** **0.245** **0.410** **0.367** **0.583** **0.608**,

**0.059** -**0.037** **0.246** **1** -**0.104** **0.050** **0.168** **0.177** **0.083** **0.147** **0.221** **0.244**,

**0.740** **0.830** **0.458** -**0.104** **1** **0.645** **0.236** **0.244** **0.298** **0.260** **0.512** **0.339**,

**0.697** **0.661** **0.609** **0.050** **0.645** **1** **0.281** **0.253** **0.442** **0.290** **0.469** **0.307**,

**0.250** **0.150** **0.338** **0.168** **0.236** **0.281** **1** **0.328** **0.519** **0.567** **0.284** **0.452**,

**0.365** **0.236** **0.245** **0.177** **0.244** **0.253** **0.328** **1** **0.237** **0.471** **0.147** **0.290**,

**0.326** **0.326** **0.410** **0.083** **0.298** **0.442** **0.519** **0.237** **1** **0.403** **0.341** **0.391**,

**0.225** **0.259** **0.367** **0.147** **0.260** **0.290** **0.567** **0.471** **0.403** **1** **0.276** **0.269**,

**0.665** **0.507** **0.583** **0.221** **0.512** **0.469** **0.284** **0.147** **0.341** **0.276** **1** **0.376**,

**0.446** **0.509** **0.608** **0.244** **0.339** **0.307** **0.452** **0.290** **0.391** **0.269** **0.376** **1**

};

NTHRESH=Ncol(thresh);

NCAT=NTHRESH+**1**;

NITEM=Nrow(LOAD);

NFACT=Ncol(LOAD);

POLYR=LOAD*FACCOR*T(LOAD);print polyr;**run**;

do j=**1** to NITEM;

POLY[j,j]=**1**;

end;

DIFFPOLY=POLY-POLYR;

Print NTHRESH[label="Number of Thresholds"], NITEM[label="Number of items"],

NCAT[label="Number of response categories"], NFACT[label="Number of factors"],

THRESH[label="Response Thresholds"],LOAD[label="Factor Loadings"],

FACCOR[label="Factor Correlation Matrix"],

POLY[label="Polychoric Correlation Matrix among Ordinal Items"] ;

print "The matrix below is the difference between polychoric correlation matrix generated

by factors and inputted polychoric correlation matrix. Nonzero values should

represent the estimated correlated errors, as specified by the user, or an error in inputted data.";

print DIFFPOLY[label=" "];

/* Computing numerator and denominator of Equation 21 */

sumnum=**0**;

addden=**0**;

do j=**1** to NITEM;

do jp=**1** to NITEM;

sumprobn2=**0**;

addprobn2=**0**;

do c=**1** to NTHRESH;

do cp=**1** to NTHRESH;

sumrvstar=**0**;

do k=**1** to NFACT;

do kp=**1** to NFACT;

sumrvstar=sumrvstar+LOAD[j,k]*LOAD[jp,kp]*FACCOR[k,kp];

end;

end;

sumprobn2=sumprobn2+probbnrm(THRESH[j,c],THRESH[jp,cp],sumrvstar);

addprobn2=addprobn2+probbnrm(THRESH[j,c],THRESH[jp,cp],POLY[j,jp]);

end;

end;

sumprobn1=**0**;

sumprobn1p=**0**;

do cc=**1** to NTHRESH;

sumprobn1=sumprobn1+CDF('NORMAL',THRESH[j,cc]);

sumprobn1p=sumprobn1p+CDF('NORMAL',THRESH[jp,cc]);

end;

sumnum=sumnum+(sumprobn2-sumprobn1*sumprobn1p);

addden=addden+(addprobn2-sumprobn1*sumprobn1p);

end;

end;

reliab=sumnum/addden;

print sumnum[label="Numerator of Equation 21"],

addden[label="Denominator of Equation 21"],

reliab[label="Nonlinear SEM Reliability Coefficient"];

/***********the following part is for the 9 weeks data*******************/

**proc** **iml**;

RESET fuzz;

THRESH={

-**2.590** -**1.503** -**0.264** **0.665**,

-**2.281** -**1.189** -**0.286** **0.582**,

-**3.012** -**2.001** -**0.826** **0.331**,

-**2.596** -**1.528** -**0.276** **0.613**,

-**1.754** -**0.331** **0** **0**,

-**1.364** **0.115** **0** **0**,

-**2.861** -**2.024** -**0.710** **0**,

-**3.106** -**1.754** -**0.153** **1.307**,

-**2.942** -**1.134** **0.233** **0**,

-**2.550** -**1.748** -**0.580** **0.284**,

-**2.516** -**0.779** **0** **0**,

-**3.079** -**2.880** -**1.674** -**0.480**

};

LOAD={

**0.825** **0** **0**,

**0.745** **0** **0**,

**0.786** **0** **0**,

**0.835** **0** **0**,

**0** **0.587** **0.402**,

**0** **0** **0.963**,

**0** **0.744** **0**,

**0** **0.532** -**0.525**,

**0** **0** **0.941**,

**0** **0.474** **0.338**,

**0** **0.785** **0**,

**0** **0.580** **0**

};

FACCOR={**1** **0.732** **0.416**,**0.732** **1** **0.625**,**0.416** **0.625** **1**};

/***********this is to remember the order of the items

/* 1 P5STRAL

2 P5STRB

3 P5STRF

4 P5STRT

5 P5C

6 P5H

7 P5K

8 P5L

9 P5PU

10 P5R

11 P5T

12 P5W

/*************************************************************/

/******POLY matrix from Mplus output******/

POLY={

**1** **0.580** **0.707** **0.667** **0.422** **0.278** **0.440** **0.139** **0.332** **0.419** **0.308** **0.397**,

**0.580** **1** **0.498** **0.664** **0.479** **0.347** **0.362** **0.143** **0.335** **0.470** **0.297** **0.311**,

**0.707** **0.498** **1** **0.620** **0.387** **0.273** **0.531** **0.181** **0.289** **0.377** **0.387** **0.390**,

**0.667** **0.664** **0.620** **1** **0.465** **0.331** **0.415** **0.173** **0.333** **0.516** **0.376** **0.337**,

**0.422** **0.479** **0.387** **0.465** **1** **0.743** **0.645** **0.021** **0.715** **0.645** **0.736** **0.456**,

**0.278** **0.347** **0.273** **0.331** **0.743** **1** **0.403** -**0.146** **0.907** **0.599** **0.554** **0.309**,

**0.440** **0.362** **0.531** **0.415** **0.645** **0.403** **1** **0.127** **0.394** **0.374** **0.623** **0.471**,

**0.139** **0.143** **0.181** **0.173** **0.021** -**0.146** **0.127** **1** -**0.164** -**0.074** **0.150** **0.133**,

**0.332** **0.335** **0.289** **0.333** **0.715** **0.907** **0.394** -**0.164** **1** **0.594** **0.489** **0.343**,

**0.419** **0.470** **0.377** **0.516** **0.645** **0.599** **0.374** -**0.074** **0.594** **1** **0.436** **0.313**,

**0.308** **0.297** **0.387** **0.376** **0.736** **0.554** **0.623** **0.150** **0.489** **0.436** **1** **0.474**,

**0.397** **0.311** **0.390** **0.337** **0.456** **0.309** **0.471** **0.133** **0.343** **0.313** **0.474** **1**

};

NTHRESH=Ncol(thresh);

NCAT=NTHRESH+**1**;

NITEM=Nrow(LOAD);

NFACT=Ncol(LOAD);

POLYR=LOAD*FACCOR*T(LOAD);print polyr;**run**;

do j=**1** to NITEM;

POLY[j,j]=**1**;

end;

DIFFPOLY=POLY-POLYR;

Print NTHRESH[label="Number of Thresholds"], NITEM[label="Number of items"],

NCAT[label="Number of response categories"], NFACT[label="Number of factors"],

THRESH[label="Response Thresholds"],LOAD[label="Factor Loadings"],

FACCOR[label="Factor Correlation Matrix"],

POLY[label="Polychoric Correlation Matrix among Ordinal Items"] ;

print "The matrix below is the difference between polychoric correlation matrix generated

by factors and inputted polychoric correlation matrix. Nonzero values should

represent the estimated correlated errors, as specified by the user, or an error in inputted data.";

print DIFFPOLY[label=" "];

/* Computing numerator and denominator of Equation 21 */

sumnum=**0**;

addden=**0**;

do j=**1** to NITEM;

do jp=**1** to NITEM;

sumprobn2=**0**;

addprobn2=**0**;

do c=**1** to NTHRESH;

do cp=**1** to NTHRESH;

sumrvstar=**0**;

do k=**1** to NFACT;

do kp=**1** to NFACT;

sumrvstar=sumrvstar+LOAD[j,k]*LOAD[jp,kp]*FACCOR[k,kp];

end;

end;

sumprobn2=sumprobn2+probbnrm(THRESH[j,c],THRESH[jp,cp],sumrvstar);

addprobn2=addprobn2+probbnrm(THRESH[j,c],THRESH[jp,cp],POLY[j,jp]);

end;

end;

sumprobn1=**0**;

sumprobn1p=**0**;

do cc=**1** to NTHRESH;

sumprobn1=sumprobn1+CDF('NORMAL',THRESH[j,cc]);

sumprobn1p=sumprobn1p+CDF('NORMAL',THRESH[jp,cc]);

end;

sumnum=sumnum+(sumprobn2-sumprobn1*sumprobn1p);

addden=addden+(addprobn2-sumprobn1*sumprobn1p);

end;

end;

reliab=sumnum/addden;

print sumnum[label="Numerator of Equation 21"],

addden[label="Denominator of Equation 21"],

reliab[label="Nonlinear SEM Reliability Coefficient"];

**Parent-Infant Caregiving Scale (PICS)**

| **How often do you find yourself doing each of the following things with your baby?** *(Please circle one response)* | | | | | |
| --- | --- | --- | --- | --- | --- |
|  | **Never** | **Rarely** | **Sometimes** | **Often** | **A lot** |
| I hold my baby | 1 | 2 | 3 | 4 | 5 |
| I pick my baby up | 1 | 2 | 3 | 4 | 5 |
| I talk to my baby | 1 | 2 | 3 | 4 | 5 |
| I cuddle my baby | 1 | 2 | 3 | 4 | 5 |
| I rock my baby | 1 | 2 | 3 | 4 | 5 |
| I kiss my baby | 1 | 2 | 3 | 4 | 5 |
| I stroke my baby’s tummy | 1 | 2 | 3 | 4 | 5 |
| I stroke my baby’s back | 1 | 2 | 3 | 4 | 5 |
| I stroke my baby’s face. | 1 | 2 | 3 | 4 | 5 |
| I stroke my baby’s arms or legs | 1 | 2 | 3 | 4 | 5 |
| I watch my baby | 1 | 2 | 3 | 4 | 5 |
| I leave her/him to lie down  (e.g. in pram / cot / basket / on mat) | 1 | 2 | 3 | 4 | 5 |
