# Supplementary figures and images for: Psychometric Properties of the Parent-Infant Caregiving Touch Scale
Source: Front Psychol. 2015 Dec 15;6:1887. doi: 10.3389/fpsyg.2015.01887 (PMC4678235; doi:10.3389/fpsyg.2015.01887)

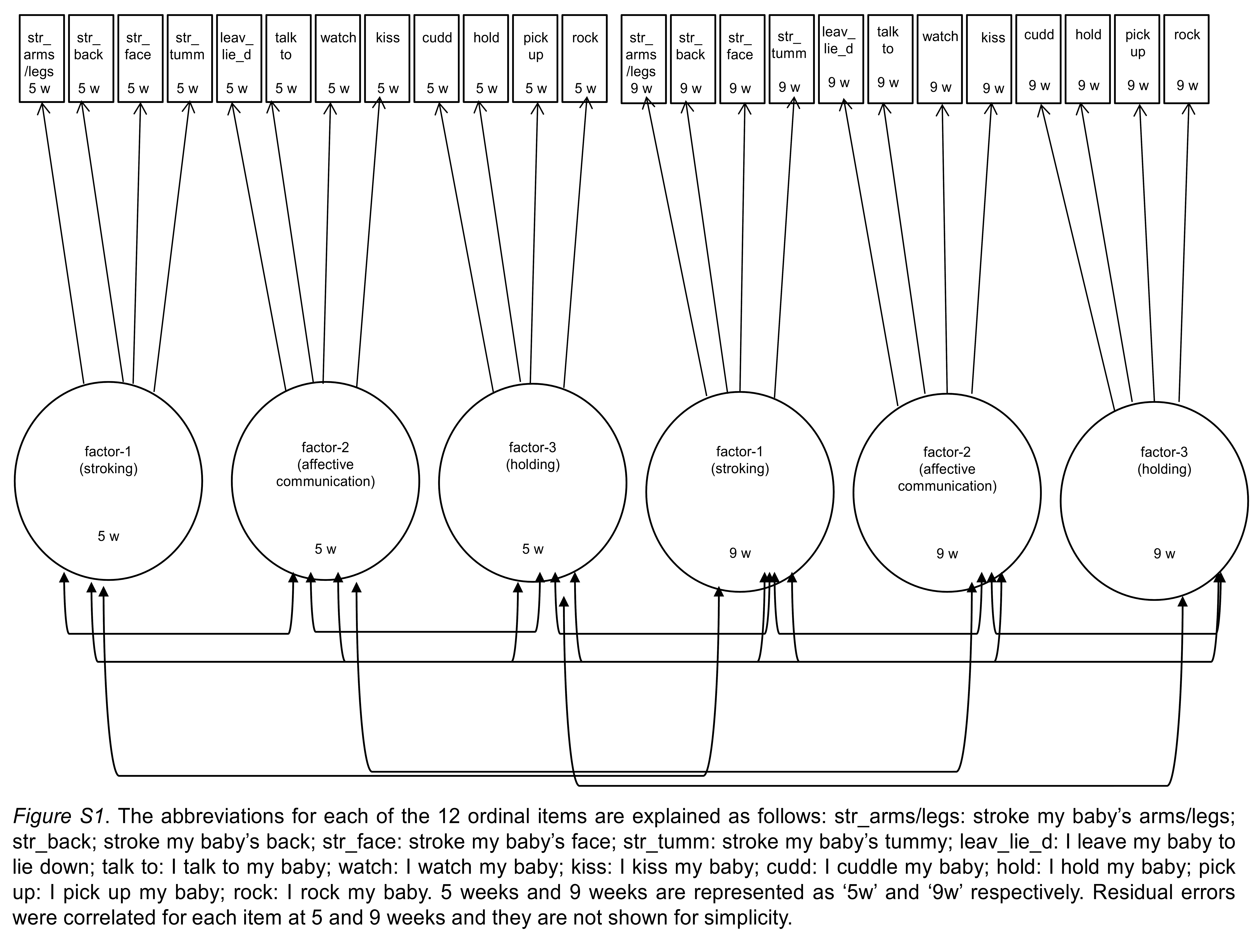

Supplement: Supplementary file 2 [file Image1.tif]
